# Modifiable Etiological Factors and the Burden of Stroke from the Rotterdam Study: A Population-Based Cohort Study

**DOI:** 10.1371/journal.pmed.1001634

**Published:** 2014-04-29

**Authors:** Michiel J. Bos, Peter J. Koudstaal, Albert Hofman, M. Arfan Ikram

**Affiliations:** 1 Department of Epidemiology, Erasmus Medical Center, Rotterdam, The Netherlands; 2 Department of Neurology, Erasmus Medical Center, Rotterdam, The Netherlands; 3 Department of Radiology, Erasmus Medical Center, Rotterdam, The Netherlands; Leiden University Medical Center, Netherlands

## Abstract

**Background:**

Stroke prevention requires effective treatment of its causes. Many etiological factors for stroke have been identified, but the potential gain of effective intervention on these factors in terms of numbers of actually prevented strokes remains unclear because of the lack of data from cohort studies. We assessed the impact of currently known potentially modifiable etiological factors on the occurrence of stroke.

**Methods and Findings:**

This population-based cohort study was based on 6,844 participants of the Rotterdam Study who were aged ≥55 y and free from stroke at baseline (1990–1993). We computed population attributable risks (PARs) for individual risk factors and for risk factors in combination to estimate the proportion of strokes that could theoretically be prevented by the elimination of etiological factors from the population.

The mean age at baseline was 69.4 y (standard deviation 6.3 y). During follow-up (mean follow-up 12.9 y, standard deviation 6.3 y), 1,020 strokes occurred. The age- and sex-adjusted combined PAR of prehypertension/hypertension, smoking, diabetes mellitus, atrial fibrillation, coronary disease, and overweight/obesity was 0.51 (95% CI 0.41–0.62) for any stroke; hypertension and smoking were the most important etiological factors. C-reactive protein, fruit and vegetable consumption, and carotid intima-media thickness in combination raised the total PAR by 0.06. The PAR was 0.55 (95% CI 0.41–0.68) for ischemic stroke and 0.70 (95% CI 0.45–0.87) for hemorrhagic stroke.

The main limitations of our study are that our study population comprises almost exclusively Caucasians who live in a middle and high income area, and that risk factor awareness is higher in a study cohort than in the general population.

**Conclusions:**

About half of all strokes are attributable to established causal and modifiable factors. This finding encourages not only intervention on established etiological factors, but also further study of less well established factors.

*Please see later in the article for the Editors' Summary*

## Abstract 2

Using data from the Rotterdam study, Michiel Bos and colleagues estimate the proportion of strokes that are attributable to established modifiable etiological factors for stroke.

*Please see later in the article for the Editors' Summary*

## Editors' Summary

**Background:**

Every year, 15 million people worldwide have a stroke. About 6 million of these people die within hours, and another 5 million are left disabled. Stroke occurs when the brain's blood supply is suddenly interrupted by a blood vessel in the brain being blocked by a blood clot (ischemic stroke) or bursting (hemorrhagic stroke). Deprived of the oxygen normally carried to them by the blood, the brain cells near the blockage die. The symptoms of stroke depend on which part of the brain is damaged but include sudden weakness or paralysis along one side of the body, vision loss in one or both eyes, and trouble speaking or understanding speech. Anyone experiencing these symptoms should seek immediate medical attention because prompt treatment can limit the damage to the brain. In the longer term, post-stroke rehabilitation can help overcome the disabilities caused by stroke, and various drugs alongside behavioral counselling can reduce the risk of a second stroke.

**Why Was This Study Done?:**

Fifty years ago, it was discovered that treatment of high blood pressure (hypertension) reduces the risk of stroke among people with severe hypertension. This discovery led researchers to search for other potentially modifiable etiological factors for stroke (risk factors that cause stroke). The list of established etiological factors now includes smoking, diabetes, atrial fibrillation (an irregular heartbeat), heart disease, and overweight/obesity, in addition to hypertension. But how many strokes would modification of these causal risk factors prevent? In this population-based cohort study, the researchers calculate the individual and combined population attributable risks (PARs) for these established etiological factors to provide an estimate of what proportion of strokes could theoretically be prevented by optimal treatment of known etiological factors. A population-based cohort study enrolls a group of people, determines their characteristics at baseline, and follows them to see whether specific characteristics are associated with specific outcomes. A PAR of an etiological factor for a disease indicates the proportion of that disease in the population that would not occur in the absence of the risk factor.

**What Did the Researchers Do and Find?:**

The researchers used data from 6,844 participants in the Rotterdam Study, which was designed to investigate the causes and consequences of long-term and disabling diseases in the elderly. At baseline, all of the participants were over 55 years old and free from stroke. During follow-up, 1,020 strokes occurred among the participants. Using data on exposure at baseline to various etiological factors for stroke, the researchers calculated PARs for individual factors and used a special statistical technique to calculate PARs for the factors in combination. The combined PAR of prehypertension/hypertension, smoking, diabetes, atrial fibrillation, heart disease, and overweight/obesity was 0.51 for any stroke. That is, about half of the strokes in the study population were attributable to this combination of etiological factors. Hypertension and smoking were the most important individual factors (PARs of 0.36 and 0.16, respectively). Notably, the inclusion of several less well established etiological factors (increased blood levels of C-reactive protein, low fruit and vegetable consumption, and thickening of the lining of arteries) only raised the total PAR for any stroke by 0.06.

**What Do These Findings Mean?:**

These findings indicate that about half of the strokes in the study cohort were attributable to established modifiable etiological factors and could theoretically be prevented by eliminating these risk factors from the population. Previous studies have estimated that a larger proportion of strokes could be prevented by eliminating known etiological factors. The researchers acknowledge that some aspects of their study may have led to an underestimation of the proportion of stroke attributable to established etiological factors and note that their findings may not be generalizable to underprivileged or racially diverse populations. Nevertheless, they argue that previous studies are likely to have overestimated the PARs for stroke because they were based on case–control studies (in which exposure to etiological factors was assessed after a stroke had occurred in cases and control individuals, rather than before a stroke as in a population-based cohort study) and often did not use optimal statistical techniques to calculate the total PAR. Importantly, these new findings underscore the importance of interventions targeted at reducing smoking and hypertension and support the search for additional etiological factors for stroke.

**Additional Information:**

Please access these websites via the online version of this summary at http://dx.doi.org/10.1371/journal.pmed.1001634.

- The US National Institute of Neurological Disorders and Stroke provides information about all aspects of stroke (in English and Spanish); its Know Stroke site provides educational materials about stroke prevention, treatment, and rehabilitation including personal stories (in English and Spanish); the US National Institutes of Health SeniorHealth website has additional information about stroke
- The Internet Stroke Center provides detailed information about stroke for patients, families, and health professionals (in English and Spanish)
- The UK National Health Service Choices website also provides information about stroke for patients and their families, including personal stories
- MedlinePlus has links to additional resources about stroke (in English and Spanish)
- Information about the Rotterdam Study is available
- The UK not-for-profit website Healthtalkonline provides personal stories about stroke

## Introduction

In the 1960s it was discovered that treatment of high blood pressure reduces the occurrence of stroke among persons with severe diastolic hypertension by more than 90% [1]. The observation that a relatively simple intervention prevents such a devastating disease continues to inspire researchers to search for other potentially modifiable etiological factors for stroke up to the present day.

This search has led to the identification of many other risk factors for stroke, some of which merely mark the increased risk without playing a role in the causal pathway (risk indicators), and some of which are presumably causal (etiological factors, or causal risk factors) [2],[3]. The impact of a causal risk factor on the burden of stroke is determined by the proportion of strokes in which the risk factor plays an indispensable role in the pathophysiological pathway, in other words, the proportion of strokes that would not have occurred had that risk factor not been present. This proportion is called the population attributable risk (PAR) [4]. A PAR indicates the maximum proportion of strokes that may be prevented by complete elimination of the risk factor, which is of course only relevant for etiological factors that can be modified. The PAR of an etiological factor depends on both the strength of the association between the etiological factor and the disease and on the prevalence of the risk factor.

Previously, PARs have been estimated for various individual etiological factors [3]. The proportion of strokes that can theoretically be prevented by optimal treatment of all known etiological factors for stroke cannot be calculated by simply adding up the adjusted PARs of the individual etiological factors: this will lead to an overestimation of the total PAR, and the total sum of the PARs may easily exceed 100%. To calculate a total PAR from multiple etiological factors, special statistical techniques need to be applied that allow a single case to be attributed to multiple etiological factors: as a rule, multiple (known and unknown) etiological factors need to be present for a stroke to occur, and adequate adjustment for confounding and interaction does not sufficiently correct for this [5]. To our knowledge, no previous cohort studies of stroke have reported the total PAR of multiple etiological factors combined. The reports that exist are either case–control studies [2],[6],[7] or did not use optimal statistical techniques [2]; all previous studies therefore likely overestimated the total PAR for stroke [4].

We were interested in finding out which proportion of strokes can theoretically be prevented by optimal treatment of all known etiological factors, and thereby how closely we have approached our ultimate goal of finding a modifiable cause for every occurring stroke. Therefore, we assessed the impact of potentially modifiable etiological factors on the occurrence of stroke in a large population-based cohort study among persons aged 55 y and over.

## Methods

### Ethics Statement

This study was approved by the Medical Ethics Committee of the Erasmus Medical Center. All participants gave written informed consent to participate in the study. Clinical investigations were conducted according to the principles expressed in the Declaration of Helsinki.

### Study Population

The Rotterdam Study is an ongoing prospective population-based cohort study that focuses on the causes and consequences of chronic and disabling diseases in the elderly [8]. The cohort started enrollment in 1990 and included 7,983 participants aged ≥55 y living in Ommoord, a district of the city of Rotterdam in the Netherlands (participation rate 78%).

### Assessment of Stroke

Stroke was defined according to World Health Organization criteria as a syndrome of rapidly developing clinical signs of focal (or global) disturbance of cerebral function, with symptoms lasting 24 h or longer or leading to death, with no apparent origin other than vascular [9]. History of stroke at baseline was assessed during the baseline interview and verified by reviewing medical records. After enrollment, participants were continuously monitored for incident stroke through automated linkage of the study database with medical records from general practitioners. Nursing home physicians' records and records from the general practitioners of participants who moved out of the district were checked on a regular basis as well. Additional information was obtained from hospital records. Potential strokes identified in medical records were reviewed by research physicians, and verified by an experienced stroke neurologist (P. J. K.) [10]. A stroke was subclassified as ischemic if a CT or MRI scan confirmed the diagnosis, or if indirect evidence (deficit limited to one limb or completely resolved within 72 h, atrial fibrillation in absence of anticoagulants) pointed at the stroke having an ischemic nature. A stroke was subclassified as hemorrhagic if a relevant hemorrhage was present on the CT or MRI scan. If we could not retrieve enough information to subclassify a stroke as hemorrhagic or ischemic, it was called unspecified. These classifications corresponded to ICD-10 codes I61, I63, and I64, respectively. Transient ischemic attacks or subarachnoid hemorrhages were not included. Follow-up was complete until January 1, 2012, for 97.1% of potential person-years [11].

### Selection and Measurement of Etiological Factors for Stroke

We selected etiological risk factors for stroke from the *Guidelines for the Primary Prevention of Stroke* from the American Heart Association/American Stroke Association [3]. We explain in Table 1 how we incorporated these etiological factors in our study.

**Table 1:** Relationship between the etiological factors described in the 2011 American Heart Association/American Stroke Association guidelines [3] and the etiological factors used in our study.

Etiological Factors Described in the 2011 American Heart Association/American Stroke Association Guidelines	Etiological Factors in Our Study
**Well-documented and modifiable risk factors**	
Hypertension	Blood pressure and blood-pressure-lowering medication
Cigarette smoking	Current and former light and heavy smoking
Diabetes	Diabetes mellitus
Dyslipidemia	TC/HDL ratio
Atrial fibrillation	Atrial fibrillation
Other cardiac conditions	Angina pectoris, CABG, PTCA, myocardial infarction
Asymptomatic carotid stenosis	Carotid IMT (subcohort)
Sickle cell disease	>97% Caucasians in our study population
Postmenopausal hormone therapy	Rare in our study population (≤1,3% at baseline)
Oral contraceptives	All participants aged ≥55 y at baseline in our study
Diet and nutrition	Fruit and vegetable consumption (subcohort)
Physical inactivity	No data available
Obesity and body fat distribution	BMI
**Less well-documented or potentially modifiable risk factors**	
Migraine	No data available
Metabolic syndrome	No data available on additional components
Alcohol consumption	0 units and ≥3 units of alcohol compared to 1–2 units per day (subcohort)
Drug abuse	Rare in our study population
Sleep-disordered breathing	No data available
Hyperhomocysteinemia	No data available
Elevated lipoprotein(a)	Data available only in limited subcohort
Hypercoagulability	No data available
Inflammation and infection	C-reactive protein quartiles (subcohort)

The classification into well-documented and modifiable risk factors versus less well-documented or potentially modifiable risk factors is adopted from the guidelines.

Blood pressure was measured twice in sitting position on the right arm with a random-zero sphygmomanometer. We used the average of these two measurements in the analyses to classify participants as having no hypertension (systolic ≤120 mmHg and diastolic ≤80 mm Hg), prehypertension (systolic >120 mm Hg or diastolic >80 mm Hg), stage I hypertension (systolic >140 mm Hg or diastolic >90 mm Hg), or stage II hypertension (systolic >160 mm Hg or diastolic >100 mm Hg). Use of antihypertensive medication was assessed during a home interview. Treated hypertension was considered controlled if, with treatment, the blood pressure measurement did not the fulfill criteria for stage I or II hypertension. Body mass index (BMI) measured at baseline was categorized as normal (BMI<25 kg/m^2^), overweight (25–30 kg/m^2^), or obese (>30 kg/m^2^). Smoking habits were assessed during the home interview and classified as never, former, or current. The number of pack-years of smoking was calculated by multiplying the number of cigarette packs smoked per day by the number of years smoked. The threshold between light and heavy smoking was defined as the median number of pack-years, calculated for former smokers and current smokers separately. We defined diabetes mellitus as a random or post-load glucose level of 11.1 mmol/l or higher, or use of antidiabetic medication. Atrial fibrillation was considered present when seen on an electrocardiogram during the baseline research center visit or when it was reported in medical records. History of angina pectoris was assessed with the Rose questionnaire [12]. History of percutaneous transluminal coronary angioplasty (PTCA) or coronary artery bypass graft (CABG) and history of myocardial infarction were positive if reported by the participant and confirmed by electrocardiogram or medical records. Coronary disease was defined as history of angina pectoris, myocardial infarction, PTCA, or CABG. Total cholesterol (TC), high-density lipoprotein (HDL), and C-reactive protein were measured in non-fasting baseline serum with automated enzymatic procedures [13]. Carotid intima-media thickness (IMT) was measured by longitudinal 2-dimensional ultrasound of the carotid artery [14]. Alcohol intake and fruit and vegetable consumption were assessed by means of a food frequency questionnaire in participants who were not demented at baseline [15]. Excessive alcohol intake was defined as more than 3 units (32 g) of alcohol per day. Fruit and vegetable consumption was categorized as adequate (>5 fruit and/or vegetable servings per day), low (3–5 servings per day), or very low (<3 servings per day) (one serving of fruit was 80 g, and one serving of vegetables was 77 g).

### Population for Analysis

Of all 7,983 participants who were enrolled into the Rotterdam Study, 7,717 were free from stroke at study baseline. After exclusion of participants who refused informed consent for retrieval of stroke follow-up data (*n* = 171) and of those who had incomplete data collection (no baseline research center visit) (*n* = 702), 6,844 participants could be included in the present analyses.

C-reactive protein, alcohol intake, fruit and vegetable consumption, and carotid IMT were assessed only in random subgroups of the cohort [13],[16]; alcohol intake and fruit and vegetable consumption were not assessed in participants with dementia at baseline [17]. Therefore, these factors were studied in combination in a subgroup of 3,570 participants who had complete assessment of all covariates.

### Statistical Analysis

PARs and 95% CIs were calculated with the Interactive Risk Attributable Program (US National Cancer Institute) [18]. This program estimates the PAR adjusted for confounding by
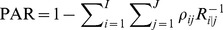
(1)where
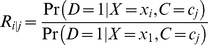
(2)and
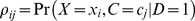
(3)with *D* = 1 denoting presence of disease, *X* denoting exposure with *i* levels, and *C* denoting a confounder with *j* levels. The relative risk is estimated from a multivariable Poisson model [19],[20].

The PAR for a combination of risk factors corresponds to the proportion of the disease that can be attributed to any of the studied risk factors. The combined PAR is not a simple summing up of the individual PARs. A disease case can simultaneously be attributed to more than one risk factor. As a result, the fraction of the case population that is attributed to or prevented by each risk factor overlaps with the fractions attributed to other risk factors. Hence, the combined PAR is usually lower than the sum of individual PARs. To estimate the proportion of the disease burden that is exclusively attributed to a specific risk factor, we calculated the combined PAR in the presence and absence of this risk factor. The difference is the “extra attributable risk,” which indicates the proportion of the disease that can be attributed exclusively to this specific risk factor [19],[21].

Missing values in the complete cohort were imputed with a linear regression model based on age and sex. No variables had >2.5% missing values. In the subcohort we did complete case analyses. Rather than dichotomizing the etiological factors, we categorized them into as many categories as possible, since this is presumed to increase the accuracy of the estimated PARs [22].

We estimated PARs for any stroke (ischemic, hemorrhagic, or unspecified) and for ischemic and hemorrhagic stroke separately. PARs for any and ischemic stroke were also assessed separately for men and women [23].

We assessed the PARs of hypertension, smoking, diabetes mellitus, atrial fibrillation, coronary disease, TC/HDL ratio, and overweight/obesity in the total cohort of 6,844 participants; we assessed the PARs of C-reactive protein, alcohol intake, fruit and vegetable consumption, and carotid IMT in the subcohort of 3,570 participants with complete assessment of those additional factors. For all strokes and ischemic strokes, we defined the lowest TC/HDL ratio quartile as the reference category, and for hemorrhagic strokes we defined the highest TC/HDL ratio quartile as the reference category, in accordance with current literature [24]. Interaction between gender and each etiological risk factor was assessed by entering interaction terms in Cox proportional hazards models, with age, gender, etiological factor, and gender×etiological factor as covariates. Statistical significance was defined at α = 0.05.

## Results

The complete study cohort comprised 6,844 participants, and the subgroup with assessment of additional characteristics comprised 3,570 participants (Table 2). The median age at start of follow-up was 68 y with interquartile range 62–76 y (mean age 69.4 y, standard deviation 6.3 y), and 60.1% of the participants were women. The participants in the subgroup with assessment of additional characteristics differed slightly from those in the complete study cohort; most notably, they were slightly younger, more often female, and smoked more.

**Table 2:** Baseline characteristics.

Characteristic	Median (Interquartile Range) or Percentage		*p*-Value for Difference^a^
	Complete Study Cohort (*n* = 6,844)	Subgroup with Data on Additional Risk Factors (*n* = 3,570)	
Age, y	68.2 (62.0–75.6)	67.7 (62.1–74.0)	<0.001
Female sex	60.1%	60.6%	<0.001
Systolic blood pressure, mm Hg	138 (123–153)	137 (123–152)	0.52
Diastolic blood pressure, mm Hg	73 (66–81)	73 (66–81)	0.68
Antihypertensive medication	12.8%	12.3%	0.56
Current smoking	22.2%	24.1%	<0.001
Former smoking	40.8%	42.3%	<0.001
Diabetes mellitus	10.3%	9.2%	0.15
Atrial fibrillation	5.0%	3.9%	0.04
Angina pectoris	3.6%	2.6%	0.42
PTCA/CABG	2.7%	1.3%	0.64
Symptomatic myocardial infarction	6.1%	6.7%	0.002
TC, mmol/l	6.6 (5.8–7.4)	6.6 (5.9–7.4)	0.01
HDL cholesterol, mmol/l	1.3 (1.1–1.6)	1.3 (1.1–1.6)	0.39
BMI, kg/m^2^	26.0 (23.8–28.4)	26.0 (23.9–28.4)	0.24
Serum C-reactive protein, mg/l	—	1.74 (0.87–3.35)	—
Alcohol intake, servings	—	0.29 (0.01–1.40)	—
Fruit and vegetable intake, servings	—	6.6 (5.1–8.2)	—
Carotid IMT, mm	—	0.77 (0.68–0.86)	—

a Difference between participants included and not included in the subgroup with data on additional risk factors. Logistic regression model adjusted for all other characteristics.

During 88,011 person-years of follow-up (mean follow-up 12.9 y, standard deviation 6.3 y), 1,020 strokes occurred, of which 610 could be classified as ischemic and 103 as hemorrhagic, and 307 remained unspecified.

When evaluated separately, the most important etiological factor for any stroke was hypertension, with a PAR of 0.36 (95% CI 0.26–0.49; Table 3). The second-ranking cause of stroke was smoking, with a PAR of 0.16 (95% CI 0.10–0.26). Also, diabetes mellitus (PAR 0.04, 95% CI 0.02–0.07) and atrial fibrillation (PAR 0.02, 95% CI 0.01–0.05) contributed significantly to the burden of stroke. Coronary disease and overweight/obesity had no statistically significant impact on the burden of stroke. The total proportion of strokes that could be attributed to any combination of these six etiological factors in combination was 0.51 (95% CI 0.41–0.62). TC/HDL ratio was not associated with the risk of stroke in the expected direction: with the quartile of participants with the lowest TC/HDL ratios as the reference category, the hazard ratios were 0.83 (95% CI 0.69–0.99) for the second quartile, 0.84 (95% CI 0.70–1.00) for the third quartile, and 0.97 (95% CI 0.82–1.16) for the fourth quartile. The participants who used cholesterol-lowering medication (prevalence 2.3%) had a slightly lower risk of stroke than those in the lowest quartile of the TC/HDL ratio distribution, but this difference was not statistically significant: the HR was 0.71 (95% CI 0.45–1.12). There was no association between HDL or non-HDL cholesterol with the risk of stroke when assessed separately (Table 4).

**Table 3:** Population attributable risks of presumed etiological factors for any stroke (*n/N* = 1,020/6,844).

Etiological Factor	Stratum	Prevalence (Percent)	Hazard Ratio (95% CI)^a^	PAR (95% CI)^a^ per Stratum	PAR (95% CI)^a^ Combined
**Hypertension**	Prehypertension	29.9	1.33 (1.06–1.67)	0.06 (0.02–0.11)	0.36 (0.26–0.49)
	Stage I	25.0	1.72 (1.38–2.16)	0.12 (0.07–0.16)	
	Stage II	14.0	2.00 (1.57–2.55)	0.09 (0.07–0.13)	
	Treated controlled	5.5	1.89 (1.37–2.59)	0.03 (0.02–0.05)	
	Treated uncontrolled	7.3	2.18 (1.66–2.86)	0.06 (0.04–0.09)	
**Smoking**	Former light	18.2	1.12 (0.93–1.36)	0.02 (0.00–0.09)	0.16 (0.10–0.26)
	Former heavy	22.6	1.30 (1.07–1.57)	0.05 (0.03–0.11)	
	Current light	9.9	1.58 (1.27–1.98)	0.04 (0.02–0.07)	
	Current heavy	12.4	1.65 (1.32–2.06)	0.05 (0.03–0.07)	
**Diabetes mellitus**	Present	10.3	1.43 (1.20–1.71)		0.04 (0.02–0.07)
**Atrial fibrillation**	Present	5.0	1.47 (1.15–1.89)		0.02 (0.01–0.05)
**Coronary disease**	Present	10.0	1.11 (0.91–1.36)		0.01 (0.00–0.08)
**BMI**	Overweight (25–30 kg/m^2^)	45.4	1.00 (0.87–1.15)	0.00 (0.00–1.00)	0.01 (0.00–0.92)
	Obese (>30 kg/m^2^)	16.6	1.07 (0.89–1.28)	0.01 (0.00–0.17)	
**Total**					0.51 (0.41–0.62)

a All analyses are adjusted for age, sex, hypertension, smoking, diabetes mellitus, atrial fibrillation, coronary disease, and overweight/obesity, if appropriate. All HRs are relative to those in whom the etiological factor is not present.

**Table 4:** Associations of HDL cholesterol and non-HDL cholesterol with stroke (*n* = 6,844).

Type of Cholesterol	Stratum	Hazard Ratio (95% CI)		
		Any Stroke (*n* = 1,020)	Ischemic Stroke (*n* = 610)	Hemorrhagic Stroke (*n* = 103)
**HDL**	Quartile 4	1 (reference)	1 (reference)	1 (reference)
	Quartile 3	0.80 (0.67–0.95)	0.86 (0.68–1.08)	0.83 (0.44–1.57)
	Quartile 2	0.88 (0.74–1.04)	0.83 (0.66–1.04)	1.46 (0.84–2.53)
	Quartile 1	0.80 (0.67–0.96)	0.73 (0.57–0.92)	0.20 (0.67–2.17)
	Treated (2.2%)	0.67 (0.43–1.05)	0.84 (0.51–1.37)	n.a.
**Non-HDL**	Quartile 1	1 (reference)	1 (reference)	1 (reference)
	Quartile 2	0.92 (0.78–1.10)	1.03 (0.81–1.30)	0.74 (0.46–1.21)
	Quartile 3	0.97 (0.82–1.16)	1.23 (0.97–1.54)	0.54 (0.32–0.92)
	Quartile 4	0.87 (0.73–1.04)	1.10 (0.86–1.38)	0.37 (0.20–0.67)

All analyses adjusted for age and sex, and computed with IBM SPSS Statistics, version 21.

n.a., not applicable because stratum without events.

There was no statistically significant interaction between gender and the studied etiological risk factors at α = 0.05. Stratified analyses for men and women separately can be found in Tables 5–8.

**Table 5:** Population attributable risks of presumed etiological factors for any stroke: men (*n/N* = 406/2,732).

Probable Etiological Factor	Stratum	Prevalence (Percent)	Hazard Ratio (95% CI)^a^	PAR (95% CI)^a^ per Stratum	PAR (95% CI)^a^ Combined
**Hypertension**	Prehypertension	31.4	1.47 (1.04–2.09)	0.09 (0.04–0.21)	0.39 (0.23–0.57)
	Stage I	24.4	1.89 (1.33–2.68)	0.13 (0.08–0.22)	
	Stage II	12.3	2.41 (1.64–3.52)	0.11 (0.07–0.16)	
	Treated controlled	6.1	1.55 (0.92–2.63)	0.02 (0.01–0.07)	
	Treated uncontrolled	7.3	1.85 (1.17–2.93)	0.04 (0.02–0.08)	
**Smoking**	Former light	19.7	1.24 (0.81–1.91)	0.04 (0.01–0.22)	0.25 (0.07–0.58)
	Former heavy	41.6	1.30 (0.87–1.94)	0.10 (0.02–0.35)	
	Current light	9.8	1.43 (0.88–2.32)	0.03 (0.01–0.11)	
	Current heavy	19.4	1.65 (1.08–2.53)	0.08 (0.03–0.17)	
**Diabetes mellitus**	Present	10.1	1.46 (1.08–1.96)		0.04 (0.02–0.10)
**Atrial fibrillation**	Present	5.6	1.27 (0.85–1.91)		0.01 (0.00–0.08)
**Coronary disease**	Present	14.9	1.19 (0.91–1.57)		0.03 (0.01–0.13)
**BMI**	Overweight (25–30 kg/m^2^)	50.0	1.14 (0.92–1.41)	0.07 (0.01–0.29)	0.07 (0.01–0.32)
	Obese (>30 kg/m^2^)	8.3	1.10 (0.76–1.59)	0.01 (0.00–0.33)	
**Total**					0.61 (0.42–0.77)

a All analyses are adjusted for age, sex, hypertension, smoking, atrial fibrillation, and diabetes mellitus, if appropriate.

**Table 6:** Population attributable risks of presumed etiological factors for any stroke: women (*n/N* = 614/4,112).

Probable Etiological Factor	Stratum	Prevalence (Percent)	Hazard Ratio (95% CI)^a^	PAR (95% CI)^a^ per Stratum	PAR (95% CI)^a^ Combined
**Hypertension**	Prehypertension	28.9	1.23 (0.91–1.66)	0.04 (0.01–0.16)	0.34 (0.20–0.51)
	Stage I	25.3	1.61 (1.20–2.16)	0.11 (0.06–0.18)	
	Stage II	15.2	1.77 (1.29–2.42)	0.08 (0.05–0.14)	
	Treated controlled	5.0	2.05 (1.37–3.06)	0.04 (0.02–0.07)	
	Treated uncontrolled	7.3	2.27 (1.61–3.20)	0.07 (0.04–0.11)	
**Smoking**	Former light	17.2	1.07 (0.85–1.35)	0.01 (0.00–0.25)	0.12 (0.07–0.20)
	Former heavy	10.0	1.37 (1.05–1.79)	0.03 (0.01–0.07)	
	Current light	9.9	1.74 (1.34–2.26)	0.05 (0.03–0.09)	
	Current heavy	7.7	1.76 (1.28–2.42)	0.03 (0.02–0.06)	
**Diabetes mellitus**	Present	10.4	1.40 (1.11–1.75)		0.04 (0.02–0.09)
**Atrial fibrillation**	Present	4.5	1.71 (1.24–2.35)		0.03 (0.01–0.06)
**Coronary disease**	Present	6.8	1.05 (0.78–1.41)		0.00 (0.00–0.65)
**BMI**	Overweight (25–30 kg/m^2^)	42.4	0.92 (0.76–1.10)	n.a.	n.a.
	Obese (BMI>30 kg/m^2^)	22.1	1.02 (0.82–1.26)	0.01 (0.00–1.00)	
**Total**					0.46 (0.33–0.60)

a All analyses are adjusted for age, sex, hypertension, smoking, atrial fibrillation, and diabetes mellitus, if appropriate.

n.a., not applicable because the hazard ratio is smaller than one.

**Table 7:** Population attributable risks of presumed etiological factors for ischemic stroke: men and women.

Probable Etiological Factor	PAR (95% CI)^a^	
	Men (*n/N* = 263/2,732)	Women (*n/N* = 347/4,112)
Hypertension	0.38 (0.20–0.60)	0.28 (0.12–0.53)
Smoking	0.24 (0.05–0.67)	0.12 (0.05–0.25)
Diabetes mellitus	0.03 (0.01–0.12)	0.03 (0.01–0.11)
Atrial fibrillation	n.a.	0.02 (0.01–0.07)
Coronary disease	0.07 (0.03–0.15)	0.00 (0.00–1.00)
Overweight/obesity	0.15 (0.05–0.37)	0.10 (0.02–0.38)
TC/HDL ratio	n.a.	0.13 (0.03–0.45)
Total	0.64 (0.41–0.82)	0.56 (0.30–0.80)

a All analyses are adjusted for age, sex, hypertension, smoking, atrial fibrillation, and diabetes mellitus, if appropriate.

n.a., not applicable because the hazard ratio is smaller than one.

**Table 8:** Population attributable risks of presumed etiological factors for which data were available only for subgroups, for any stroke: men and women.

Etiological Factor	PAR (95% CI)^a^ Combined	
	Men (*n/N* = 215/1,405)	Women (*n/N* = 330/2,165)
Previous total PAR^b^	0.74 (0.50–0.89)	0.49 (0.32–0.66)
Serum C-reactive protein	0.06 (0.00–0.85)	0.06 (0.00–0.67)
Fruit and vegetable intake^c^	0.09 (0.03–0.23)	0.00 (0.00–1.00)
Carotid IMT	0.09 (0.01–0.69)	0.16 (0.04–0.49)
Grand total	0.76 (0.54–0.90)	0.56 (0.34–0.75)

a All analyses are adjusted for age, sex, hypertension, smoking, diabetes mellitus, atrial fibrillation, coronary disease, and overweight/obesity, if appropriate.

b Total PAR based on hypertension, smoking, diabetes mellitus, atrial fibrillation, coronary disease, and overweight/obesity, calculated in this subgroup of the study population.

c Very low intake (<3 servings per day) and low intake (3–5 servings per day) versus adequate intake (>5 servings per day) of fruits and/or vegetables.

When we restricted the analyses to ischemic strokes, the PARs of the individual risk factors did not materially change (Table 9); the largest observed (non-significant) difference was for overweight/obesity, which had a PAR of 0.01 (95% CI 0.00–0.92) for any stroke and a PAR of 0.12 (95% CI 0.05–0.27) for ischemic stroke. The total PAR of hypertension, smoking, diabetes mellitus, atrial fibrillation, coronary disease, overweight/obesity, and TC/HDL ratio for ischemic stroke was 0.55 (95% CI 0.41–0.68).

**Table 9:** Population attributable risks of presumed etiological factors for ischemic stroke.

Etiological Factor	PAR (95% CI)^a^ (*n/N* = 610/6,844)
Hypertension	0.33 (0.20–0.49)
Smoking	0.16 (0.08–0.30)
Diabetes mellitus	0.03 (0.01–0.08)
Atrial fibrillation	0.00 (0.00–0.16)
Coronary disease	0.03 (0.01–0.08)
Overweight/obesity	0.12 (0.05–0.27)
TC/HDL ratio	0.03 (0.00–0.82)
Total	0.55 (0.41–0.68)

a All analyses are adjusted for age, sex, hypertension, smoking, diabetes mellitus, atrial fibrillation, coronary disease, TC/HDL ratio, and overweight/obesity, if appropriate. Category boundaries for quartiles of TC/HDL ratio were 4.38, 5.30, and 6.42 for men and 3.94, 4.78, and 5.82 for women.

When we studied hemorrhagic strokes (Table 10), smoking was the most important etiological factor (PAR 0.40, 95% CI 0.22–0.60), followed by unfavorable TC/HDL ratio (in the case of hemorrhagic stroke, we considered a low ratio to be unfavorable): PAR 0.31 (95% CI 0.11–0.63). Hypertension was third, with a PAR of 0.24 (95% CI 0.04–0.73). Overweight/obesity was not associated with the risk of hemorrhagic stroke. This resulted in a total PAR of 0.70 (95% CI 0.45–0.87) for hemorrhagic strokes attributable to hypertension, smoking, diabetes mellitus, and unfavorable TC/HDL ratio.

**Table 10:** Population attributable risks of presumed etiological factors for hemorrhagic stroke.

Etiological Factor	PAR (95% CI)^a^ (*n/N* = 103/6,844)
Hypertension	0.24 (0.04–0.73)
Smoking	0.40 (0.22–0.60)
Diabetes mellitus	0.03 (0.00–0.26)
TC/HDL ratio^b^	0.31 (0.11–0.63)
Total	0.70 (0.45–0.87)

a All analyses are adjusted for age, sex, hypertension, smoking, diabetes mellitus, and TC/HDL ratio, if appropriate.

b The quartile with the highest ratio was the reference category. Category boundaries for quartiles of TC/HDL ratio were 6.42, 5.30, and 4.38 for men and 5.82, 4.78, and 3.94 for women.

Serum C-reactive protein, fruit and vegetable intake, alcohol consumption, and carotid IMT were studied in a subcohort of 3,570 participants because of incomplete collection of data in the other participants. The combined PAR of hypertension, smoking, diabetes mellitus, atrial fibrillation, coronary disease, and overweight/obesity was slightly higher in this subcohort than in the complete cohort (0.59, 95% CI 0.47–0.71). Addition of serum C-reactive protein, low fruit and vegetable intake, and high carotid IMT raised the PAR by 0.06 to 0.65 (95% CI 0.51–0.77; Table 11). Alcohol consumption was not related with stroke; when we compared consumption of 0 units and consumption of >3 units of alcohol per day with the recommended 1 or 2 units per day, the hazard ratios were 0.99 (95% CI 0.82–1.18) and 0.91 (95% CI 0.70–1.20), respectively.

**Table 11:** Population attributable risks of presumed etiological factors for which data were available only for subgroups, for any stroke (*n/N* = 545/3,570).

Etiological Factor	Classification	Prevalence (Percent)	Hazard Ratio (95% CI)^a^	PAR (95% CI)^a^ per Classification	PAR (95% CI)^a^ Combined
**Previous total PAR** b					0.59 (0.47–0.71)
**Serum C-reactive protein**	Quartile 2	25	1.06 (0.82–1.36)	0.01 (0.00–0.58)	0.06 (0.00–0.54)
	Quartile 3	25	1.04 (0.81–1.34)	0.01 (0.00–0.87)	
	Quartile 4	25	1.13 (0.88–1.46)	0.03 (0.00–0.21)	
**Fruit and vegetable intake**	3–5 servings	20	1.21 (0.99–1.47)	0.04 (0.01–0.12)	0.04 (0.01–0.13)
	<3 servings	4	0.96 (0.60–1.55)	n.a.	
**Carotid IMT**	Quartile 2	25	1.00 (0.76–1.32)	0.00 (0.00–1.00)	0.14 (0.04–0.40)
	Quartile 3	25	1.20 (0.91–1.57)	0.05 (0.01–0.18)	
	Quartile 4	25	1.38 (1.05–1.81)	0.09 (0.04–0.20)	
**Grand total**					0.65 (0.51–0.77)

Category boundaries for C-reactive protein quartiles were 0.896, 1.900, and 3.960 for men and 0.902, 1.830, and 3.360 for women. Category boundaries for carotid IMT quartiles were 0.72, 0.80, and 0.92 for men and 0.67, 0.76, and 0.85 for women.

a All analyses are adjusted for age, sex, hypertension, smoking, diabetes mellitus, atrial fibrillation, coronary disease, and overweight/obesity, if appropriate.

b Total PAR based on hypertension, smoking, diabetes mellitus, atrial fibrillation, coronary disease, and overweight/obesity, calculated in this subgroup of the study population.

n.a., not applicable because the hazard ratio is smaller than one.

## Discussion

We found that about half of all strokes in our study cohort were attributable to established modifiable etiological factors, and could theoretically be prevented by elimination of these risk factors from the population. Less well established or potentially modifiable etiological factors did not materially change this proportion. Our estimates are considerably lower than those reported previously.

Several methodological issues need to be discussed. The strengths of our study are the meticulous stroke case finding and the nearly complete follow-up (loss of potential person-years, 2.8%). An advantage of our stringent stroke monitoring procedures was that we could include stroke patients who had not been referred to a neurologist (31% of all stroke cases). Because in these cases neuroimaging was not performed, we could subclassify only 21% of them as ischemic or hemorrhagic strokes. In contrast, 93% of stroke cases that were referred to a neurologist could be subclassified. Overall, we could not determine the subtype of stroke in 307 participants.

A statistical issue to note is that the reported lower bound of some confidence intervals of PARs were close to 0 and therefore should be interpreted cautiously. The reason is that a PAR cannot be smaller than 0, which led to some of these confidence intervals being truncated at 0.

Our study is novel in four important ways. First, previous studies on the subject were case–control studies, which may have overestimated the PARs: when risk factors are assessed after occurrence of stroke in a case–control setting, they are different than when they are measured before occurrence of stroke in a cohort study. In addition, previous studies included only strokes that were referred to a hospital, whereas we also included strokes that were not referred to a hospital (31% of all cases in our cohort). Since elderly people tend to be referred less often than younger people, and the associations between stroke and its risk factors weaken with increasing age [25], not including the non-referred strokes may lead to an overestimation of the PARs. Second, not all previous studies used statistical methods that account for the possibility that a single stroke can be attributable to multiple etiological factors [19],[20], which may have caused them to overestimate PARs. Third, some previous studies included risk factors for which the association with stroke and the causal role in the etiology are not well established, leading to an overestimation of the observed associations. Fourth, the PARs that we observed are considerably lower than most PARs reported previously, which is an important counterbalance for previous findings.

A comparison of the PARs of *individual* etiological factors in our study with those reported previously shows that the PARs of most factors tended to be slightly lower in our study than in earlier reports, with the exception of smoking, which had a similar PAR [3]. Several explanations are possible. First, selection bias may have played a role in earlier estimates, since strong associations tend to be more easily published than weak associations. Second, our study participants are aware of their cardiovascular risk status because of their participation in the study. The healthcare system in the study district is well developed and accessible to everyone without substantial financial barriers. Therefore, most participants have their risk factors treated according to up-to-date guidelines. Third, our study participants all live in a middle and high income area in the city, and >97% are Caucasians. As a result, poverty-related health problems are rare. The prevalence of, for example, alcoholism, malnourishment, diabetes, and overweight/obesity is relatively low in our study population compared to other populations. This means that our results may not be generalizable to underprivileged or racially diverse populations. Fourth, we did not dichotomize the etiological factors but categorized them, which may have resulted in more accurate estimates [22]. Fifth, all risk factors were assessed at baseline. During follow-up, which was on average 12.9 y, risk factor profiles of individual participants may have changed, which could have weakened the associations we found. Sixth, the median age at start of follow-up of our study participants was relatively high (median 68 y), and the relation between stroke and its etiological factors weakens with increasing age [25].

Only a few previous studies have reported PARs of *combinations* of classical etiological factors for stroke. An overview of these studies can be found in Table 12. All of these studies were case–control studies or were based on previous literature. The Comparative Risk Assessment project, which based the prevalence of etiological factors and their associations with stroke on previous literature, found a total PAR of 0.70–0.76 [26]. A case–control study conducted in Rochester, Minnesota, US, reported a total PAR of 0.57 (95% CI 0.48–0.67), which after inclusion of emerging risk factors increased to 0.80 [2]. The INTERSTROKE study, a case–control study in 22 countries, found a total PAR of 0.90 (95% CI 0.85–0.94) [7]. A case–control study from the São Vicente de Paulo Hospital in Osório, Brazil, reported a total PAR of 0.99 (95% CI 0.96–1.00) [6]. We estimated the total PAR of established modifiable etiological factors to be 0.51 (95% CI 0.41–0.62) for stroke, which is considerably lower than that reported in these previous studies.

**Table 12:** Studies reporting total population attributable risk for stroke.

Study	Publication Year	Design	Outcome	Included Variables	PAR (95% CI)
Comparative Risk Assessment project [26]	2003	Multiple	Stroke	High blood pressure, high cholesterol, high BMI, low fruit and vegetable intake, physical inactivity, tobacco, alcohol	0.70–0.76
Rochester [2]	2006	Case–control	Ischemic stroke	Established causal factors (hypertension, transient ischemic attack, cigarette smoking, ischemic heart disease, atrial fibrillation, diabetes mellitus, mitral valve disease); emerging novel risk factors (raised apoB/apoA 1 ratio, obesity, physical inactivity, pyschosocial stress, low fruit and vegetable intake)	0.57 (0.48–0.67); 0.80 if emerging risk factors are included
INTERSTROKE [7]	2010	Case–control	Stroke	Self-reported hypertension, smoking status, waist-to-hip ratio, diet risk score, regular physical activity, diabetes mellitus, alcohol intake, psychosocial factors, cardiac causes, ratio of ApoB to ApoA1	0.90 (0.85–0.94)
São Vicente de Paulo Hospital [6]	2012	Case–control	Ischemic stroke	Hypertension, atrial fibrillation, left ventricular hypertrophy, presence of carotid bruit, heavy smoking status, diabetes, alcohol abuse, HDL cholesterol, physical inactivity	0.99 (0.96–1.00)
Rotterdam Study (present study)	2014	Cohort	Stroke, ischemic stroke, hemorrhagic stroke	Hypertension, smoking, diabetes mellitus, atrial fibrillation, coronary heart disease, overweight/obesity, TC/HDL ratio	0.51 (0.41–0.62) for stroke; 0.55 (0.41–0.68) for ischemic stroke; 0.70 (0.45–0.87) for hemorrhagic stroke

There are several explanations why our estimates of the total PAR are lower than reported previously, in addition to the slightly lower estimates of PARs for individual risk factors. First, our study is a cohort study, whereas all previous studies that reported a total PAR were case–control studies [2],[6],[7],[26]. The latter studies could overestimate the PAR because of reverse causality (risk factors were assessed after and not before occurrence of stroke) and because strokes that were not referred to a hospital (31% of cases in our study) were not included. Second, to combine the PARs of multiple etiological factors, special statistical techniques have to be applied: with inadequate techniques (when only confounding and interaction are corrected for, and a disease case cannot simultaneously be attributed to more than one risk factor), total PARs are very likely to be overestimated and can even exceed 100% [18]. Third, the various studies selected slightly different etiological factors, as specified in Table 12. This could also have played a role in the observed differences. As mentioned, we selected the etiological factors in our study based on the 2011 American Heart Association/American Stroke Association guidelines [3] and the data that were available. We had no information on physical inactivity: it has been shown that physically active persons have a 25%–30% lower risk of stroke than the least active persons, although no intervention trials have been performed [3]. The association between physical activity and stroke is at least partly mediated by blood pressure, diabetes, and body weight [3], for which we did have data. We may have slightly underestimated the total PAR because of lack of data on physical activity. Lack of data on other factors (Table 1) probably did not materially influence our total PAR, because the causality for these factors has not been established, and either the previously found associations were weak or the factors are rare in the general population [3].

It should be kept in mind that the causal role and the modifiability of the majority of these factors have not been established and are in some instances even unlikely. For example, C-reactive protein levels are consistently associated with the risk of cardiovascular disease, but genetic studies negate the causality of the association. C-reactive protein is not really modifiable in and of itself, and presumably is merely a marker of other presently unknown risk factors. Therefore, it is difficult to modify C-reactive protein or its underlying cause [27].

The relationship between cholesterol and stroke is controversial. The most widely accepted hypothesis is that with increasing serum levels of TC and decreasing serum levels of HDL cholesterol, the risk of ischemic stroke slightly increases and the risk of hemorrhagic stroke decreases [3],[24]. Our results corroborate this view. It is possible that in our study the contrasting relationships that cholesterol has with ischemic and hemorrhagic stroke annulled the association between cholesterol and any stroke. The theoretical positive effect of cholesterol treatment per se on ischemic stroke risk may be frustrated by a negative effect on hemorrhagic stroke risk. However, trials have shown that treatment with statins prevents strokes, although it cannot be excluded that pleiotropic effects beyond cholesterol modification account for the beneficial effect of statins [28].

It should be noted that the proportion of strokes that can actually be prevented is in practice probably lower than suggested by the PAR, for several reasons. First, if a stroke is prevented successfully in a person, this person can still suffer later in life from stroke due to other causes. Second, it is unlikely that risk factors can be eliminated completely. For example, we assumed that blood pressure should be maintained below 120/80 mm Hg, but evidently it is not possible to attain such a blood pressure level in all persons. Third, we assumed that all risk factors we studied are modifiable, and that modification reverses the complete excess risk that is portended by the risk factor. This, too, is unlikely: not only because the modifiable factors may, at least partially, be markers of other factors that may not be modifiable, but also because risk factors may already have caused irreversible damage at the time that treatment is initiated.

It is uncertain whether all variation in stroke occurrence can be explained by modifiable etiological factors; we consider it likely that a large proportion of the variation in stroke occurrence can only be explained by non-modifiable factors, and even the role of chance should not be underestimated.

In conclusion, our data suggest that half of all strokes in persons aged 55 y and over might theoretically be prevented by optimal treatment or elimination of hypertension, smoking, diabetes mellitus, atrial fibrillation, coronary disease, and overweight/obesity. Our findings underscore the importance of hypertension and smoking as etiological factors for stroke, and encourage continuing interventions on these factors. On the other hand, our data suggest that a considerably larger proportion of strokes than commonly assumed cannot be prevented by interventions on currently known etiological factors, and show the importance of the search for novel etiological factors.

In our opinion, our results underscore that the battle against smoking should continue until smoking is banned entirely. In addition, the cost-effectiveness of population screening for hypertension and blood pressure treatment in medium-risk populations should be further explored, because even in our study population with 100% blood pressure awareness, hypertension is still the main cause of stroke. Easier treatment regimens might make it easier for persons with hypertension to adhere to their medication. Last, the quest for new etiological factors may identify new treatment targets. Emerging radiological techniques enable the imaging of intracranial atherosclerosis, which may lead to new ways to prevent stroke [29]. The fields of epigenomics and proteomics hold undelivered promises to identify yet unknown modifiable risk factors [2].

## Supporting Information

Checklist S1 **STROBE checklist.**
(PDF)

## Abbreviations

BMI: body mass index
CABG: coronary artery bypass graft
HDL: high-density lipoprotein
IMT: intima-media thickness
PAR: population attributable risk
PTCA: percutaneous transluminal coronary angioplasty
TC: total cholesterol
